# Surface-bound bovine serum albumin carrier protein as present in recombinant cytokine preparations amplifies T helper 17 cell polarization

**DOI:** 10.1038/srep36598

**Published:** 2016-11-03

**Authors:** Lei Dong, Alexandra Helmke, Ari Waisman, Hermann Haller, Andreas Pich, Sibylle von Vietinghoff

**Affiliations:** 1 Department of Internal Medicine, Division of Nephrology and Hypertension, Hannover Medical School, Hannover, Germany; 2 Department of Nephrology, Tongji Hospital, Huazhong University of Science and Technology, China; 3 Institute for Molecular Medicine, University of Mainz, Germany; 4 Institute for Toxicology, Proteomics Unit, Hannover Medical School, Hannover, Germany

## Abstract

Understanding of T helper 17 lineage (T_H17_) polarization has been significantly promoted by cell culture experiments that reduce the complexity of the *in vivo* environment. We here investigated T_H17_ amplification by coating of cytokine preparations. Cytokine preparations coated to the surface compared to the same amount given in solution significantly enhanced T_H17_ polarization assessed by flow cytometry and interleukin (IL)-17A, IL-17F and RORγt mRNA expression. T cell proliferation and T_H1_ polarization were similarly enhanced while T_REG_ polarization was impeded. T_H17_ amplification was replicated by coating the plate with low amounts of FCS or albumin as used as carrier protein for cytokines (0.5 μl 0.1%). It was unaltered by filtration, protein digestion and arylhydrocarbon receptor blockade, not replicated by LPS and independent of integrin stimulation. T_H17_ amplification required anti-CD3 stimulation and was T cell intrinsic. Supernatants of CD4^+^ cells polarized on coated cytokine preparations with carrier albumin conferred amplification to fresh splenocytes. Coating markedly elevated CD4^+^ IL-22 mRNA expression and IL-22 blockade significantly reduced T_H17_ amplification. Our data show T_H17_ amplification by coated albumin in the low amounts present in recombinant cytokine preparations. This unexpected adjuvant like effect underscores the need for controls also for temporal and spatial factors in cell culture.

*In vitro* culture is a standard method to investigate mechanisms of T helper cell polarization and efficacy of therapeutic interventions targeting T helper cell subsets^1^,^2^,^3^. T cells are activated by stimulation through T cell receptor (TCR) interactions with cognate major histocompatibility complex molecules and co-stimulation via CD28^4^.

Polarization to specific T helper lineages requires cytokines in addition to T cell receptor stimulation. Transforming growth factor beta (TGFβ), interleukin (IL)-6 and IL-23 promote murine T helper 17 cell lineage (T_H17_) polarization^5^,^6^,^7^,^8^. STAT3 and RORγt transcription factors promote T_H17_ signature cytokine IL-17A and IL-17F gene expression^9^. STAT3 can be activated by IL-21 and IL-22, a member of the IL-10 family. Both IL-21 and IL-22 are expressed in T_H17_ cells under specific conditions^10^,^11^,^12^,^13^,^14^,^15^.

In addition to cytokines, a number of other agents modulate T_H17_ polarization^1^,^2^,^3^. For example, low molecular weight ligands to the aryl hydrocarbon receptor (AhR) are found in high concentrations in Iscove’s modified Dulbecco’s (IMDM) medium and are therefore common cell culture ingredients^16^,^17^. AhR activation induces a marked increase in T_H17_ cell proportion and cytokine production^18^,^19^,^20^. Lipopolysaccharide (LPS), a component of gram-negative bacteria, is a common contaminant of recombinant protein preparations^21^. Its role in T_H17_ cell polarization is controversial. While high concentrations increased T_H17_ polarization *in vitro*^22^ and *in vivo*^23^, a lower concentration was without effect^24^. Enhancement of T cell proliferation is the intended effect of vaccine adjuvants *in vivo*, some of which directly stimulate T cells^25^. Beyond enhancing antigen specific response, some adjuvants favor distinct T_H_ lineages, for example, alum induces an innate response that promotes T_H2_ polarization^26^,^27^. Regarding T_H17_ polarization^28^, complete Freund’s adjuvant (CFA), a water-in-oil emulsion with heat-killed mycobacteria induces IL-17 secreting cells *in vivo.* The *in vitro* effect has not been reported.

Interaction with the vascular wall and other surfaces, for example via integrins and activation of the cytoskeleton modifies T cell response^29^. Beyond this, integrins can promote T_H17_ differentiation by binding an RGD peptide sequence in TGFβ^30^,^31^. Fractalkine, the unique ligand of CX3CR1, is a stalked cytokine that exists in soluble and surface bound form *in vivo* and modulates immune cell migration and function^32^. Fractalkine effects *in vitro* have largely been studied using surface-bound recombinant cytokine^33^,^34^,^35^. Fractalkine receptor CX3CR1 is expressed on T cells^36^ including T_H1_ cells^37^,^38^. We recently demonstrated its expression on both T_H17_ and T_REG_ cells and induction by TGFβ during lymphocyte culture^39^. This led us to investigate the effect of coated and soluble recombinant fractalkine in T_H17_ cell polarization. Performing controls with specific receptor deficient cells revealed a receptor-unspecific T_H17_ amplification loop by diverse coated versus soluble recombinant cytokine preparations.

To define appropriate controls for further T_H17_ polarization experiments, where specific gene deficient controls might not be available, we here explored the underlying mechanism.

## Results

### Amplification of T_H17_ polarization by a coated fractalkine preparation is receptor independent

**Figure 1:**
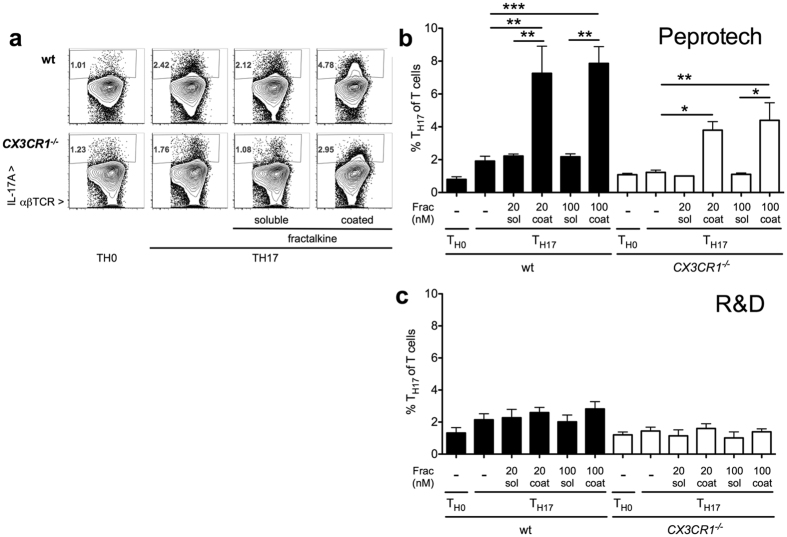
Effect of coated versus soluble fractalkine preparations on T_H17_ cell polarization in CX3CR1^−/−^ cells. (**a–c)** Wildtype and fractalkine receptor deficient (*CX3CR1*^*−/−*^) cells were subjected to T_H17_ polarization with IL-6, TGFβ and IL-23 in the absence and presence of coated (“coat”) and soluble (“sol”) recombinant fractalkine at 20 and 100 nM final concentrations. The proportion of T_H17_ cells was assessed by intracellular IL-17A staining after re-stimulation on day 4 (**a**, examples, **b** statistical analysis of fractalkine from Peprotech, and **c**, from R&D Systems, n = 4–8 from 2–4 indep. exp.).

Given the effect of fractalkine receptor CX3CR1 on T cell polarization demonstrated by others^37^ and impeded T_H17_ polarization in specific gene deficient cells found by us^39^, we investigated the effect of recombinant fractalkine on T_H17_ polarization *in vitro*. Coated recombinant fractalkine preparation from one, but not another vendor markedly increased T_H17_ polarization (Fig. 1a–c). However, this effect was also observed in *CX3CR1*^*−/−*^ cells included as a specificity control, suggesting an unspecific effect of coated substance. Similarly, a coated, but not soluble IL-17A preparation markedly enhanced T_H17_ polarization of both wildtype and IL-17 receptor A deficient (*Il17ra*^*−/−*^) splenocytes (Suppl. Fig. 1A). Again, this effect appeared only for one of the tested preparations.

This marked receptor-independent effect led us to further investigate its mechanism in order to avoid unspecific findings.

### Cytokine coating to the cell culture surface amplifies T_H17_ and T_H1_, but not T_REG_ polarization

**Figure 2:**
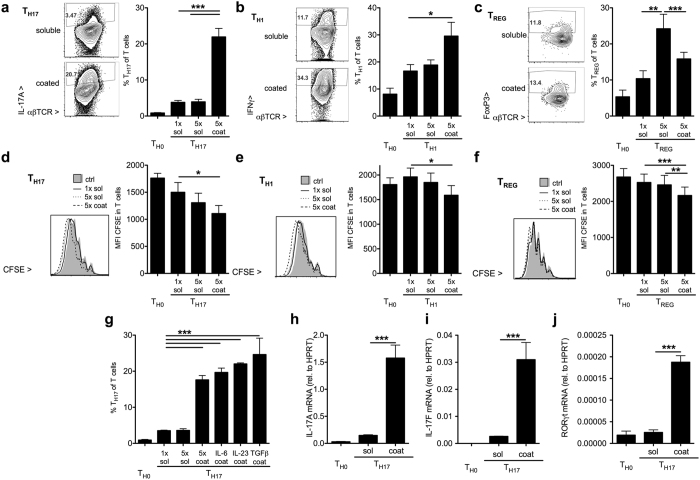
Recombinant cytokine preparations coated to the cell culture plate increase T cell proliferation and amplify T_H17_ and T_H1_, but not T_REG_ polarization. For T helper cell polarization in total splenocytes on anti-CD3 and anti-CD28 antibodies, cytokines were either added to the cell culture medium in soluble form (“sol”) at standard or elevated concentration or coated to the cell culture vessel beforehand (“coat”) (see methods and Suppl. Fig. 6 for details). **(a–c)** T helper cell polarization was assessed by intracellular staining for IL-17A (T_H17_, A, n = 14, 7 exp.), IFN-γ (T_H1_, B, n = 6, 3 exp.) after restimulation on day 4 of polarization with IL-6 (50 ng/ml), TGFβ (1 ng/ml), IL-23 (20 ng/ml) or 10 ng/ml IL-12, respectively, or 5x these amounts as indicated. T_REG_ polarization was assessed by staining for FoxP3 on day 3 of polarization with TGFβ (10 ng/ml) and IL-2 (10 ng/ml) or 5x these amounts as indicated. (C, n = 10, 5 exp., examples in A-C are 1x soluble and 5x coated experiments). **(d–f)** Proliferation was assessed by CFSE dilution in T_H17_ (**d**), T_H1_ (**e**) and T_REG_ (**f**) polarized cells (n = 6 each, 3 indep. exp.). **(g)** The effect of coating with individual cytokines was investigated on day 4 of T_H17_ polarization (n = 4, 2 indep. exp.). **(h–j)** Expression of IL-17A (**h**) and IL-17F (**i**) and the T_H17_ signature transcription factor RORγt (**j**) was measured by qPCR on day 4 of polarization (n = 4, 2 indep. exp.).

We investigated the effect of coating of cytokine preparations used for polarization of T_H1_, T_H17_ and T_REG_ cells in parallel (see methods and Suppl. Fig. 6 for detailed protocols). Pre-coating the plate with T_H17_ polarizing cytokines IL-6, IL-23 and TGFβ amplified T_H17_ polarization compared to the same amount given to the cell culture medium (Fig. 2a). A similar effect was observed for T_H1_ polarization (Fig. 2b). In contrast, T_REG_ polarizing agents in coated form decreased the proportion of T_REG_ cells (Fig. 2c). In parallel, coated but not soluble IL-17A inhibited both wildtype and *Il17ra*^*−/−*^ T_REG_ polarization (Suppl. Fig. 1B). T cell proliferation assessed by CFSE dilution was increased by coating in all tested conditions (Fig. 2d–f). There was no effect of coating on the proportion of live T cells among all events recorded after restimulation in either T_H17_ or T_REG_ conditions, while a minor decrease in the T_H1_ condition was noted (Suppl. Fig. 2A–C). However, the proportion of live cells among all T cells was not affected for either lineage (Suppl. Fig. 2D,E). T_H17_ polarization induced a significant increase in CD44 and loss of CD62l, this was however not significantly altered by coating (Suppl. Fig. 3).

Amplification of T_H17_ polarization required all T_H17_ polarizing cytokines TGFβ, IL-6 and IL-23 (data not shown), but was obtained if any single one of them was coated to the plate while the others were in solution (Fig. 2g). T_H17_ polarization was further investigated by qPCR. Pre-coated preparations significantly increased IL-17A, IL-17F and RORγt gene expression during T_H17_ polarization (Fig. 2h–j). Similarly, in *CX3CR1*^*−/−*^ cells, coating with a recombinant fractalkine preparation enhanced RORγt in T_H17_ cells, with much less effect on Tbet and FoxP3 (Suppl. Fig. 4). T_H2_ marker transcription factor GATA3 and cytokines IL-4 and IL-5 were not significantly affected in the tested lineages (Suppl. Fig. 5).

These data depict an application dependent, non-cytokine-specific amplification of T_H17_ cells by commercial cytokine preparations.

### Recombinant cytokine preparations amplify T_H17_ polarization in an adjuvant like fashion

Information on cytokine sources and specification is summarized in Suppl. Table 1. We contacted all suppliers for a complete list of ingredients and their concentrations, however, did frequently not obtain complete information. LPS is a known contaminant of recombinant cytokines. A large dose range that included values markedly above the maximal doses claimed for the preparations (below 1 or 0.1EU/μg) was tested in T_H17_ polarization (Suppl. Figs 6 and 7A). Neither coated nor soluble LPS affected the polarization. Also, integrin stimulation with collagen or RGD peptide had no effect (Suppl. Fig. 7B).

**Figure 3:**
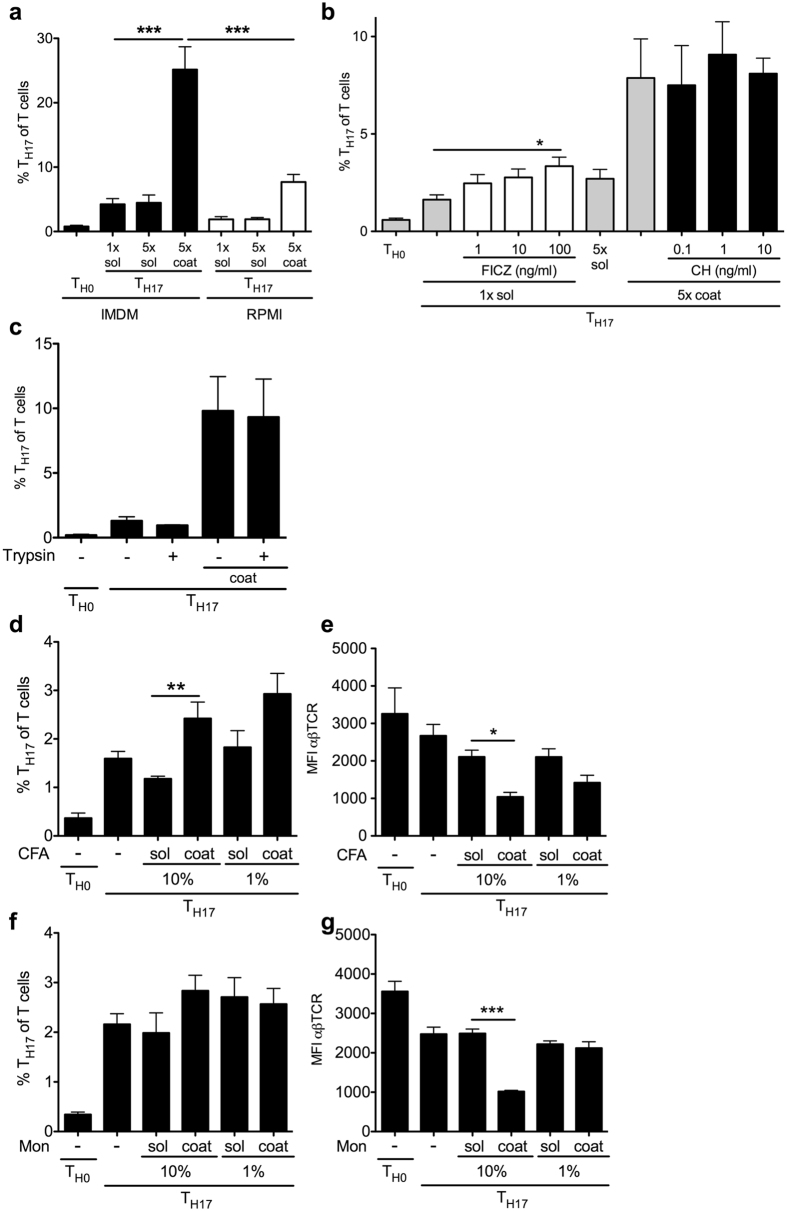
T_H17_ amplification is independent of AhR stimulation and also observed after culture on coated CFA. **(a)** T_H17_ polarization with and without cytokine coating in AhR ligand rich (IMDM) versus low (RPMI) cell culture media for 4 days (n = 8 from 4 indep. exp., Bonferroni after ANOVA). **(b)** T_H17_ polarization with soluble cytokine in the presence of coated AhR agonist FICZ at the indicated doses and antagonist CH-223191 on coated cytokine preparation (n = 6 from 3 indep. exp., Bonferroni of selected conditions after ANOVA). **(c)** Cell culture plate coating with T_H17_ polarizing cytokines was performed with or without trypsin digestion as described in methods. (4 days, n = 4 from 2 indep. exp.). (**d–g)** CFA (**d,e**) and Montanide (Mon, **f,g**) water-in-oil adjuvants were added in solution or coated to the plate during T_H17_ polarization and the proportion of T_H17_ cells (**d,f**) and the mean TCR expression on live T cells (**e,g**) determined (4 days, n = 6–8, 3–4 indep. exp., Bonferroni after ANOVA). For all T_H17_ polarizations cytokines were added at standard concentration (50 ng/ml IL-6, 1 ng/ml TGFβ, 20 ng/ml IL-23) to the media of all cells, coating with 5x of these concentrations was as indicated in panels A and B, all cells were restimulated with PMA/ionomycin.

AhR ligands that are abundant in IMDM compared to RPMI favor T_H17_ polarization^16^. When the effect of coating was tested in direct comparison of both media, the proportion of T_H17_ cells was higher in IMDM. However, the effect of coated versus soluble preparations was observed in both (Fig. 3a). This additive effect argues against a direct AhR stimulation by components of the cytokine preparations. More specifically, AhR agonist FICZ in coated form dose dependently increased T_H17_ polarization, but did not reach the level observed for coated cytokine preparations. Conversely, AhR blocker CH-223191 did not impair T_H17_ amplification by coated preparations (Fig. 3b).

Proteome analysis of an IL-17A preparation that amplified T_H17_ in coated, but not soluble form, compared to one that did not, showed mostly plasma and keratin components in both and no obvious candidates for T_H17_ polarization (Suppl. Table 2). Functionally, trypsin digestion of the coated proteins did not abrogate T_H17_ amplification by coating (Fig. 3c) while it significantly decreased soluble cytokine polarizing function (Suppl. Fig. 7D). These effects are consistent with an adjuvant effect. CFA is one of the strongest known adjuvants with a known T_H17_ favoring function *in vivo*^40^,^41^,^42^. *In vitro*, CFA enhanced T_H17_ polarization in coated, but not soluble form (Fig. 3d), albeit to a somewhat lesser degree than coated cytokine preparations. In direct comparison, a new generation of water-in-oil emulsion adjuvant, Montanide ISA51VG, was investigated^43^. Coated Montanide did not significantly enhance T_H17_ polarization (Fig. 3f). Both adjuvants, however, induced much stronger TCR downregulation in coated than soluble form (Fig. 3e,g), a feature of combined CD3 and T cell receptor stimulation^44^.

These results indicate an adjuvant-like amplification of T_H17_ polarization by coated cytokine preparations.

### T_H17_ amplification requires anti-CD3 stimulation and is replicated by coating with albumin

**Figure 4:**
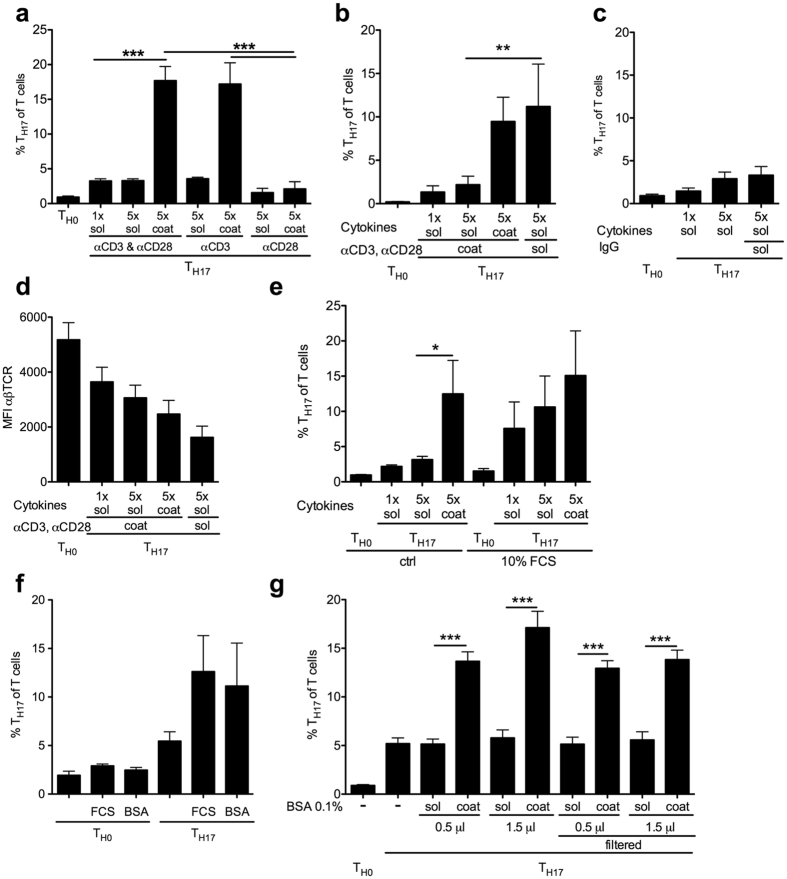
Anti-CD3 antibody in conjunction with FCS or albumin amplifies T_H17_ polarization. T_H17_ polarization with coated versus soluble cytokine preparations in the absence and presence of anti-CD3 and anti-CD28 antibodies (n = 6, 3 exp., Bonferroni after ANOVA). **(b)** Addition of anti-CD3 and anti-CD28 together with coated and soluble preparations (n = 6, 3 indep. exp., Bonferroni after ANOVA). **(c)** Addition of IgG isotype (n = 6, 3 indep. exp., Bonferroni after ANOVA). **(d)** αβTCR expression on the T cell surface after T_H17_ polarization with addition of anti-CD3 and anti-CD28 antibodies together with cytokines (IL-6, IL-23 and TGFβ) in solution or coated form (n = 6 from 3 indep. exp.). **(e)** Pre-adsorption of the cell culture plate with 10% FCS (n = 4, 2 indep. exp., Bonferroni after ANOVA). **(f)** Effect of coating cell culture grade FCS (0.5 μl 10% in PBS) or 0.1% BSA (0.5 μl 0.1%, n = 4, 2 indep. exp). **(g)** Coating with 0.5 μl and 1.5 μl 0.1% BSA as used as carrier protein with and without an additional filtration step (0.2 μm, n = 8, 4 indep. exp., Bonferroni after ANOVA). For T_H17_ polarizations, cytokines were added at 1x concentration (50 ng/ml IL-6, 1 ng/ml TGFβ, 20 ng/ml IL-23) to the media of all cells, coating with 5x of these concentrations is indicated in the legends, polarizations were conducted for 4 days and all cells were restimulated with PMA/ionomycin.

To study the role of T cell receptor stimulation in the T_H17_ amplification caused by cytokine preparations, we next investigated the protocol of anti-CD3 and anti-CD28 stimulation. As a standard, these antibodies were coated to the culture plate (Suppl. Fig. 6). T_H17_ amplification depended on anti-CD3 but not anti-CD28 antibody (Fig. 4a). It was preserved with a different ultrapure anti-CD3 preparation (n = 2, data not shown). Giving anti-CD3 antibody and cytokines simultaneously into solution was equivalent to coating both to the plate (Fig. 4b). A rat IgG isotype to the anti-CD3 antibody was used as control. It did not amplify T_H17_ polarization (Fig. 4c). T cell receptor (TCR) surface expression significantly decreased with cytokine coating and also simultaneous addition of cytokines and anti-CD3 antibody (Fig. 4d). As a standard, we used highly adsorbent tissue culture plasticware and therefore hypothesized that pre-adsorbed anti-CD3 molecule interaction with T cells and cytokine preparations might be limited. Indeed, previous surface blocking with FCS (Fig. 4e) and use of sterile low absorbent polystyrene and polypropylene flow cytometry tubes (data not shown) rendered coated and soluble cytokine preparations equipotent in T_H17_ amplification. On the other hand, these results might suggest that spatial proximity or even direct interaction of the anti-CD3 antibody with a component of coated FCS was amplifying T_H17_ differentiation. Albumin is a main part of serum. Indeed, also lower amounts of coated FCS (0.5 μl 10% in PBS) and low amounts of albumin (BSA) significantly amplified T_H17_ polarization (Fig. 4f). This range of BSA was present as a carrier protein in the recombinant cytokine preparations. While coated BSA in the amount of the cytokine itself did not alter T_H17_ polarization (Suppl. Fig. 7D), 0.5 μl of 0.1% BSA as used as carrier indeed amplified T_H17_ polarization in coated but not soluble form (Fig. 4g). T_H17_ amplification persisted after an additional filtration step (Fig. 4g). Thus, coated albumin completely replicated the receptor independent effects of coated cytokine preparations that initially prompted our study (Fig. 1, Suppl. Fig. 1).

These results identify coated albumin as amplifier of T_H17_ polarization and are consistent with a combined effect of albumin and the anti-CD3 antibody.

### T_H17_ amplification is T cell intrinsic and induces IL-22 production

**Figure 5:**
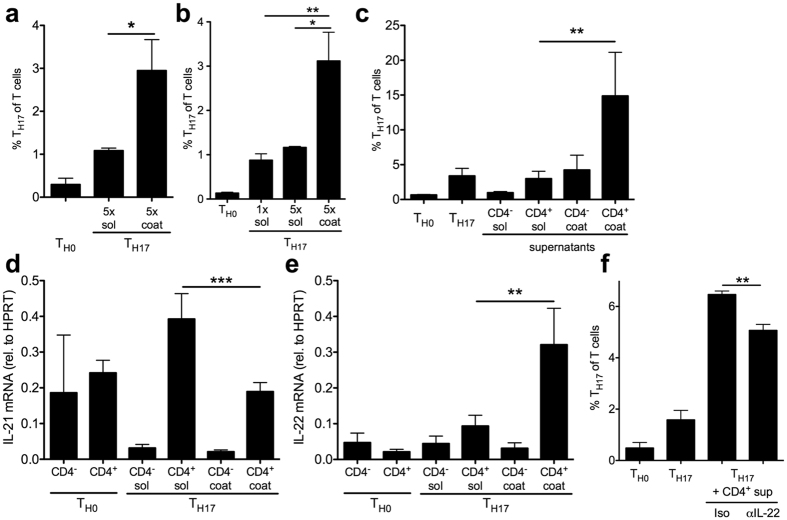
T_H17_ amplification by coated cytokine preparations containing albumin carrier is T cell intrinsic and induces IL-22. **(a,b)** T_H17_ polarization of CD4^+^ (A) and naïve CD4^+^ T cells (B) on coated and with soluble cytokine preparations (n = 4, 2 indep. exp. each). **(c)** T_H17_ polarization of splenocytes cultured with the supernatants of CD4^+^ enriched or CD4^+^ depleted splenocytes that had been stimulated with either coated or soluble T_H17_ cytokines (n = 8, 4 indep. exp., Bonferroni after ANOVA). **(d,e)** Expression of IL-21 (D) and IL-22 (**e**) was measured by qPCR on day 3 of culture of CD4^+^ and CD4^−^ enriched splenocytes without exogenous cytokines (T_H0_) and with either coated or soluble T_H17_ polarizing cytokines (n = 6, 3 indep. exp.). **(f)** Anti-IL-22 antibody or isotype control was applied to T_H17_ polarization of splenocytes stimulated with supernatants from CD4^+^ cells grown on coated T_H17_ polarizing cytokines (n = 4, 2 indep. exp., T_H17_ polarizations were conducted for 4 days, cytokines were added at 1x concentration (50 ng/ml IL-6, 1 ng/ml TGFβ, 20 ng/ml IL-23) to the media of all cells, coating with 5x of these concentrations is indicated in the legend of panel A–E, cells were restimulated with PMA/ionomycin for flow cytometric analysis).

To investigate whether the observed effect was T cell intrinsic, magnetically enriched CD4^+^ splenocytes were polarized with coated or soluble cytokines containing carrier albumin. The T_H17_ amplification conveyed by coating persisted (Fig. 5a), also with a negative selection method from a different manufacturer (data not shown) and also in negatively selected naïve CD4^+^ T cells (Fig. 5b). To determine whether T cells secreted a soluble factor that amplified their own polarization, we treated splenocytes during T_H17_ polarization with supernatants from CD4^+^ and CD4^−^ cells that had been cultured with either soluble or coated cytokines. Supernatants from CD4^+^ much more than CD4^−^ splenocytes cultured on coated cytokines conferred T_H17_ amplification (Fig. 5c). These data are consistent with a CD4^+^ autocrine loop enhancing T_H17_ polarization.

We next investigated IL-21 and IL-22, cytokines that can be produced by T_H17_ cells and can both activate STAT3, a T_H17_ enhancing transcription factor^14^,^15^. While IL-21 gene expression was decreased in CD4^+^ cells on coated cytokine preparations compared to cytokines in solution, IL-22 was significantly upregulated (Fig. 5d,e). To test for a function of secreted IL-22, T_H17_ polarization was performed in the presence of supernatant of CD4^+^ cells polarized on coated cytokines as in Fig. 5c, but with blockade of IL-22 or isotype control (Fig. 5f). IL-22 receptor mRNA expression was detectable in splenocytes after culture on coated preparations (0.06 ± 0.00% of HPRT, n = 2, data not shown). Indeed, IL-22 blockade significantly reduced T_H17_ amplification by supernatants of CD4^+^ T cells stimulated with coated cytokine preparations.

This indicates that IL-22 contributes to T_H17_ amplification by coated cytokine preparations containing carrier albumin.

## Discussion

Our data show significant amplification of *in vitro* T_H17_ polarization if low amounts of albumin or cytokine preparations containing carrier albumin are coated to the plate in conjunction with anti-CD3 antibody. The amplification was mediated in an autocrine fashion and also increased IL-22 production.

T_H17_ polarization required IL-6, IL-23 and TGFβ cytokines in any of the tested conditions, but was markedly enhanced if these or the carrier protein only were coated to the plate. Enhancement of T_H17_ polarization required combined action of the anti-CD3 antibody and albumin or a cytokine preparation containing it, possibly, but not necessarily in coated form. In addition, coating induced T cell proliferation and a marked loss of TCR surface expression in all studied conditions. This is consistent with TCR activation^44^ and TCR-CD3 complex formation that promotes T cell proliferation^45^.

The vaccine adjuvant CFA in coated form similarly enhanced T_H17_ polarization, together with downregulation of TCR surface expression. Beyond amplification of the immune response, some vaccine adjuvants favor individual T helper cell lineages^25^. For CFA, *in vivo* measurements have demonstrated elevated IL-17A, IL-10 and IL-22 cytokine expression in CD4^+^ T cells under T_H17_ polarizing conditions^40^. Also, an early report before discovery of IL-17A describes a marked increase in myelopoiesis, a hallmark of IL-17A induced G-CSF production, after CFA treatment^46^. The effect of coated albumin or carrier containing cytokine preparations in amplifying T_H17_ cells was rather stronger than for CFA in our experiments. Also, the albumin effects were completely resistant to digestion with trypsin, and indeed, understanding of mechanisms of most vaccine adjuvants functions is incomplete at present^25^.

At the same time of T_H17_ and T_H1_ amplification, T_REG_ polarization was impaired by coated cytokine preparations containing albumin carrier. While T_H17_ and T_REG_ cells share a common requirement of TGFβ for polarization^47^, a high TGFβ concentration induces the T_REG_ defining transcription factor FoxP3 that represses RORγt^47^,^48^. A large number of other factors regulating gene expression including mammalian target of rapamycin (mTOR), hypoxia inducible factor 1 alpha (HIF1α) and retinoic acid receptor alpha (RORα) are also involved in this reciprocal regulation^1^,^2^,^3^. T_REG_ cells limit response to vaccines, for example the effectiveness of BCG vaccination against *M. tuberculosis*^49^. Therefore, adjuvants favoring T_H1_ and T_H17_ at the cost of T_REG_ cells might be helpful. However, decreasing T_REG_ function has a strong deleterious potential and needs to be tested with great caution^50^.

IL-22 was increased during T_H17_ polarization on coated cytokines containing albumin carrier and was at least partly responsible for autocrine amplification, possibly by STAT3 activation^14^. TGFβ suppresses IL-22 expression^51^ at the concentration of 1 ng/ml that is commonly used for T_H17_ polarization and also in our experiments^47^. This restriction was apparently removed in cells on coated cytokine preparations. Similar mechanisms may contribute to IL-17A and IL-22 co-expression *in vivo*^52^,^53^,^54^. IL-22 is highly relevant biologically. For example, it promotes wound healing in a large range of conditions^13^,^14^,^15^,^55^. In other settings, co-expression of IL-22 together with IL-17A promoted airway^53^ and chronic liver inflammation and fibrosis in hepatitis B virus infected patients and HBV transgenic mice^56^ and expression of antimicrobial peptides in human keratinocytes^54^. Our data indicate that direct IL-22 effects also need to be considered when *in vitro* polarized T_H17_ cells are adoptively transferred to investigate their roles in disease models *in vivo*.

In summary, our data show that T_H17_ polarization is significantly amplified and IL-22 expression increased by a combined action of coated albumin or cytokine preparations containing it as carrier and anti-CD3 antibody that can be inadvertently caused by *in vitro* culture conditions. This adjuvant-like effect on T_H17_ amplification adds a new degree of complexity to T_H17_ polarization *in vitro* and possibly alters T_H17_ function in immune disorders *in vivo*.

## Methods

### Animals

Wild-type (wt) C57Bl/6, *CX3CR1*^*−/−*^ ( = *CX3CR1*^*gfp/gfp *^57) (Jackson Labs, Bar Harbor, ME) and *Il17ra*^*−/−*^ mice^52^ all on C57Bl/6 background, were genotyped by PCR. Mice were kept in specific-pathogen-free conditions. Harvest of primary murine cells was approved by the Landesamt für Verbraucherschutz und Lebensmittelsicherheit, Lower Saxony, Germany according to the current regulations. All methods were performed according to the relevant guidelines.

### Cell culture, stimulation and T cell polarization

T cell culture was performed in 96 well plates (Nunclon Delta Surface, Thermo Fisher Scientific, Waltham, MA, USA), unless sterile polystyrene (Falcon, Thermo Fisher Scientific) or polypropylene (Sarstedt, Nürmbrecht, Germany) tubes were used as indicated. Cultures were in complete IMDM (Gibco, Thermo Fisher Scientific) with 10% fetal calf serum (FCS, PAN Biotech, Aidenbach, Germany) and penicillin (10,000 U/ml)/streptomycin (10,000 μg/ml) (Gibco, Waltham, MA) unless otherwise indicated.

Total and CD4^+^ enriched or depleted (CD4 L3T4, MicroBeads, Miltenyi, Bergisch Gladbach, Germany or MojoSort^TM^ Mouse CD4 T cell isolation kit, Biolegend, San Diego, CA, USA, each applied according to the manufacturer’s instructions, reaching above 90% purity) mouse splenic lymphocytes were cultured at a concentration of 5 × 10^6^/ml. MojoSort^TM^ Mouse CD4 Naïve T Cell Isolation Kit (Biolegend, San Diego, CA, USA) was used as directed by the manufacturer. Cultures in complete IMDM without exogenous cytokines are depicted as T_H0_. T_H17_ polarization was performed with anti-IFN-γ and anti-IL-4 (both 3 μg/ml) and IL-6 (50 ng/ml), TGFβ (1 ng/ml), IL-23 (20 ng/ml) (all Biolegend) added in 100 μl medium, unless otherwise indicated. For T_H1_ polarization, 10 ng/ml IL-12 and anti-IL-4 (3 μg/ml, both Biolegend) was added. For T_REG_ polarization, culture was in RPMI with TGFβ (10 ng/ml, Biolegend) and IL-2 (10 ng/ml, Peprotech, Rocky Hill, USA). If indicated, 5x these cytokine concentrations were used.

Coating to the cell culture dish was performed for at least 30 min at 37 °C. Coating with purified anti-CD28 (1 μg/ml, clone 37.51) and anti-CD3 leaf (10 μg/ml, clone 17A2) or anti-CD3 ultraleaf (10 μg/ml, clone 145–2C11, all Biolegend) was in a total volume of 7 μl/well. For soluble antibody addition, 1 μg/ml anti-CD28 and 10 μg/ml anti-CD3 were added with the medium. Leaf-IgG anti-CD3 isotype (Biolegend) was given precoated or soluble at a concentration of 10 μg/ml. If indicated, collagen (2 μl, 0.1% stock solution) and RGD (2 μl, 10 mg/ml stock solution) (both Sigma-Aldrich, St. Louis, MI, USA), FCS (10 μl, 10%, in sterile pyrogen free PBS (Lonza, Basel, Switzerland)), bovine serum albumin (BSA, Sigma-Aldrich, St. Louis, MI) dissolved in sterile PBS at a concentration of 10 ng/ μl and 0.1% = 10 μg/ μl and filtered (0.2 μm) as indicated, LPS at the indicated concentrations (500000 EU/mg, Escherichia coli O111:B4, Sigma-Aldrich), trypsin (to reach a protease:protein ratio of 1:5–1:20 (w/w) in PBS, Serva Electrophoresis, Heidelberg, Germany), CFA (1 μl of 10% and 1% V/V in PBS as indicated, Sigma-Aldrich) or Montanide ISA 51VG (1 μl of 10% and 1% V/V in PBS as indicated, Elaiapharm, Paris, France) was added to the coating step or in solution. CH-223191 (5 mg/ml in DMSO) and FICZ (1 mg/ml in DMSO, both Sigma-Aldrich) were further diluted in PBS and used in the indicated amount^51^. Final DMSO concentrations ranged from 1:500 to 1:1,000,000, a range that did not significantly affect the T_H17_ polarization in our setting (Suppl. Fig. 8). CX3CL1 (1 μg/ml, 1.7 μl or 8.5 μl for final concentrations of 20 and 100 nM, Peprotech and R&D Systems (Minneapolis, MN, USA)), and 5 μl IL-17A (1 μg/ml to reach a final concentration of 50 ng/ml, Miltenyi and R&D Systems) were used pre-coated or in the same amount applied together with medium as indicated above. Coated and soluble cytokine and antibody addition is also detailed in Suppl. Fig. 6.

Cell culture supernatants from CD4^+^ and CD4^−^ enriched cells were harvested on day 3 without restimulation and added at 1:1 with fresh medium and soluble T_H17_ polarizing cytokines at standard concentration to splenocytes for 4 days cell culture. If indicated, anti-IL-22 or isotype (polyclonal Goat IgG, final concentration: 2.5μg/ml, R&D Systems, Minneapolis, MN, USA) was applied. CFSE (Life technologies, Darmstadt, Germany) was used according to the manufacturer’s instructions. Re-stimulation before intracellular cytokine staining was with 10 ng/ml PMA and 500 ng/ml ionomycin (both from Sigma-Aldrich) as described^39^.

### Flow cytometry

The following antibodies were used: TCRβ (H57-597), FoxP3 (150D), IL-17A (TC11-18H10.1), IFN-γ (XMG1.2), CD44 (IM7), CD62l (MEL-14), CD69 (H1.2F3) (Biolegend and eBioscience, San Diego, CA, USA). Near-infrared LIVE/DEAD^®^ Fixable Dead Cell Stain Kit (Invitrogen, Carlsbad, CA), Foxp3/Transcription Factor Staining Buffer set (eBioscience) and Fixation/Permeabilization Solution Kit (BD Biosciences) were used according to the manufacturer’s instructions. Flow cytometry analysis was performed on a Becton-Dickinson FACS Canto. Data were analyzed using FlowJo software (Tree Star Inc., Ashland, OR). Gating was performed for live, TCRβ^+^ cells before analysis of IL-17A, IFN-γ and FoxP3 expression as % of parent.

### RNA isolation and real time PCR

RNA was isolated using NucleoSpin® RNAII or NucleoSpin® RNA Plus Kit (Macherey-Nagel, Duren, Germany) and RNA yield and purity determined with a Colibri Microvolume Spectrometer (Titertek-Berthold, Pforzheim, Germany). After reverse transcription (M-MLV-RT, Promega, Mannheim, Germany), real-time PCR was performed on a LightCycler^®^480 using SYBR-Green (FastStart Taq DNA Polymerase dNTPack, Roche, Grenzach-Wyhlen, Germany). Primers were as follows (5′-3′): HPRT: FP: CAGTCCCAGCGTCGTGATTA, RP: AGCAAGTCTTTCAGTCCTGTC, *Il4:* FP: GGTCTCAACCCCCAGCTAGT RP: GCCGATGATCTCTCTCAAGTGAT, *Il5:* FP: CTCTGTTGACAAGCAATGAGACG RP: TCTTCAGTATGTCTAGCCCCTG, *Il10:* FP: GCTCTTACTGACTGGCATGAG RP: CGCAGCTCTAGGAGCATGTG, *Il17a*: FP: TTTAACTCCCTTGGCGCAAAA, RP: CTTTCCCTCCGCATTGACAC, *Il17f*: FP: TGCTACTGTTGATGTTGGGAC, RP: AATGCCCTGGTTTTGGTTGAA, *Il21*: FP: GGGGACAGTGGCCCATAAATC, RP: GTGCCCCTTTACATCTTGTGG, *Il22*: FP: ATGAGTTTTTCCCTTATGGGGAC, RP: GCTGGAAGTTGGACACCTCAA, *Il22r*: FP: ATGAAGACACTACTGACCATCCT, RP: CAGCCACTTTCTCTCTCCGT, *Rorgt*: FP: CCGCTGAGAGGGCTTCAC, RP: TGCAGGAGTAGGCCACATTAC, *Tbet:* FP: CAACAACCCCTTTGCCAAAG RP: TCCCCCAAGCAGTTGACAGT, *Foxp3:* FP: ACTGGGGTCTTCTCCCTCAA RP: CGTGGGAAGGTGCAGAGTAG, *Gata3:* FP: CTCGGCCATTCGTACATGGAA RP: GGATACCTCTGCACCGTAGC. Data were analyzed with HPRT as a reference gene using LinRegPCR software^58^.

### Proteome analysis of recombinant murine IL-17A preparations

Proteins were separated by SDS-PAGE, gel pieces were destained, dehydrated and digested with 5 ng/μl trypsin (37 °C, 300rpm) after rehydration. Extracted peptides were dried via vacuum centrifugation and separated using a nanoflow reversed phase chromatography system (RSLC, Thermo Fisher Scientific, Germany). For shotgun analysis, peptides enriched on the trap column were eluted with a multistep linear gradient connected to the nano electrospray source of an LTQ orbitrap velos (Thermo Fisher Scientific, Germany) for shotgun or a 4000 Qtrap (AB Sciex, Germany) mass spectrometer. After ionisation using a metal-coated fused silica emitter, most intensive ions according to overview scans were submitted to CID fragmentation. MS data were processed with MaxQuant software (Version 1.2.0.18). MS-spectra were searched in the SwissProt/Uniprot database with a false discovery rate of 0.01 at protein and peptide level as described^59^.

### Statistical analysis

Two-tailed student t-test was used to compare two conditions. If more than two conditions were compared, Bonferroni’s test of selected conditions was applied after ANOVA. P-values <0.05 were considered significant. Data are expressed as mean ± SEM. P values are indicated as follows: *p < 0.05, **p < 0.01, ***p < 0.001.

## Additional Information

**How to cite this article**: Dong, L. *et al*. Surface-bound bovine serum albumin carrier protein as present in recombinant cytokine preparations amplifies T helper 17 cell polarization. *Sci. Rep.* **6**, 36598; doi: 10.1038/srep36598 (2016).

**Publisher’s note:** Springer Nature remains neutral with regard to jurisdictional claims in published maps and institutional affiliations.

## Supplementary Material

Supplementary Information

Supplementary Table 2
