# Supplementary material for: Surface-bound bovine serum albumin carrier protein as present in recombinant cytokine preparations amplifies T helper 17 cell polarization
Source: Sci Rep. 2016 Nov 3;6:36598. doi: 10.1038/srep36598 (PMC5093436; doi:10.1038/srep36598)
Supplement: Supplementary Information [file srep36598-s1.pdf]

Surface-bound bovine serum albumin carrier protein as present in recombinant cytokine preparations amplifies T helper 17 cell polarization

Lei Dong, Alexandra Helmke, Ari Waisman, Hermann Haller, Andreas Pich, and Sibylle von Vietinghoff

Supplemental figures and table

Suppl. figure 1

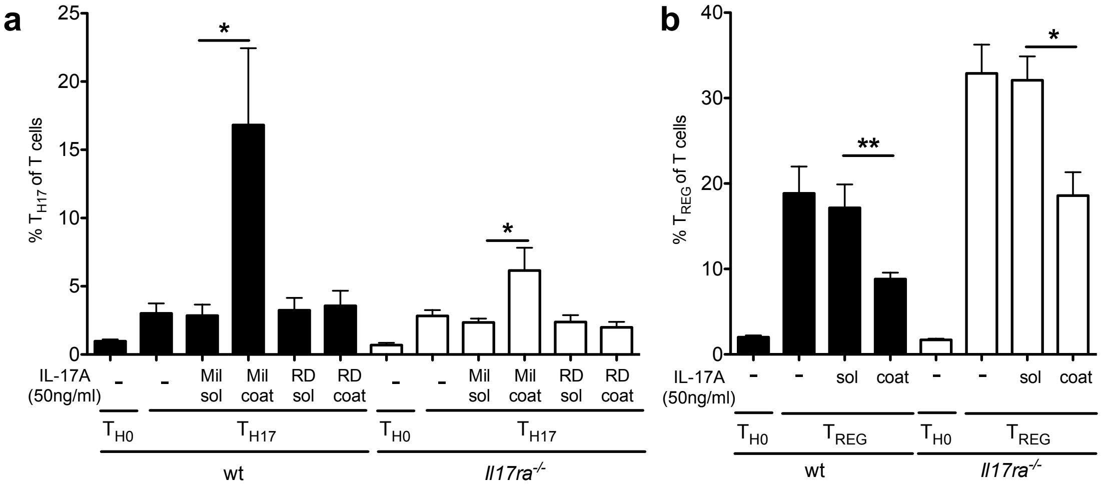

Supplemental figure 1: Effect of coated vs. soluble IL-17A preparations on T_{H17} and T_{REG} cell polarization in $IL17ra^{-/-}$ cells

(A,B) Wildtype and interleukin 17 receptor A deficient (*$Il17ra^{-/-}$*) cells were subjected to T_{H17} and T_{REG} polarization in the absence and presence of coated ("coat") and soluble ("sol") recombinant IL-17A preparations from 2 different suppliers (Miltenyi Biotech, "Mil", and R&D systems, "R&D") at a final concentration of 50ng/ml. The proportion of T_{H17} cells was assessed by intracellular IL-17A staining after restimulation on day 4 (A, n=8 from 4 indep. exp.). The proportion of T_{REG} cells was investigated after intracellular staining for Foxp3 on day 3 of culture with IL-17A (Miltenyi Biotech, B, n=8 from 4 indep. exp. each).

Suppl. figure 2

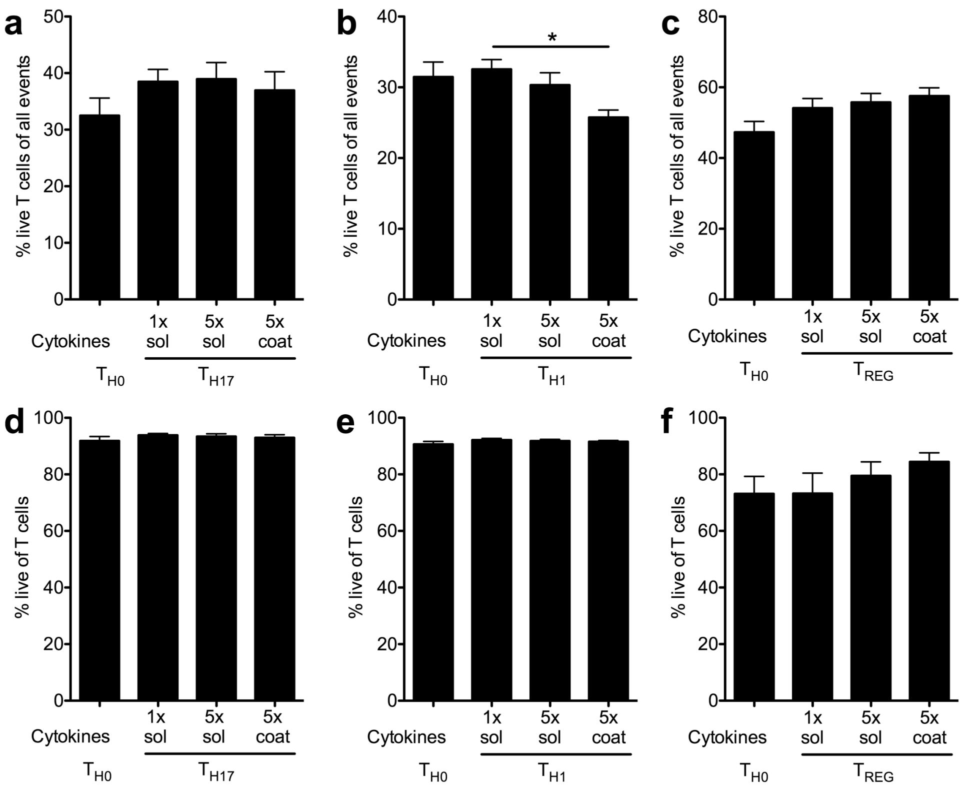

Supplemental figure 2: T cell viability

T helper cell viability of the cultures depicted in figure 2 was assessed after restimulation on day 4 of T_{H17} **(A,D),** T_{H1} **(B,E)** and day 3 of T_{REG} (**C,F**, no restimulation) polarization. **(A-C)** Live T cells expressed as % live, $\alpha\beta TCR^+$ of all events. **(D-F)** Live as % of all T cells (n= 6 from 3 independent experiments for each subtype, Bonferroni after ANOVA).

Suppl. figure 3

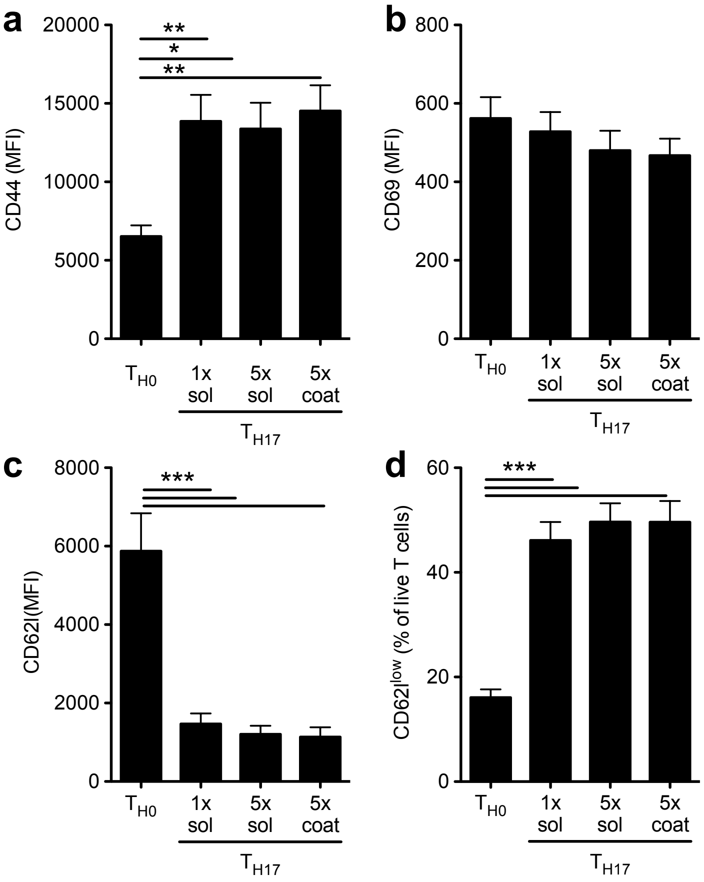

Supplemental figure 3: Effect of coating on activation marker expression after T_{H17} polarization

T_{H17} cells were polarized for 4 days with coated or soluble IL-6 (50ng/ml), TGFβ (1ng/ml), IL-23 (20ng/ml) or 5x these amounts as indicated and surface expression of CD44 (A), CD69 (B) and CD62l (C) as well as the proportion of CD62l^{low} cells (D) analyzed among live T cells (Bonferroni after ANOVA, n=10 from 5 exp.).

Suppl. figure 4

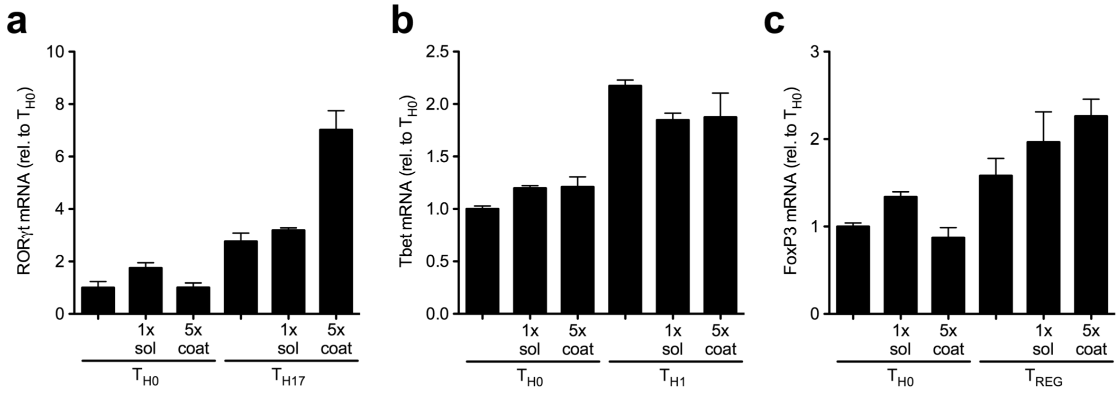

Supplemental figure 4: Lineage marker transcription factor expression with coated and soluble cytokine preparation

(A-C) Fractalkine receptor deficient (*CX3CR1*$^{-/-}$) cells were subjected to T_{H17} polarization in the absence and presence of coated ("coat") and soluble ("sol") recombinant fractalkine at 100nM final concentration. mRNA expression of RORγt (A), Tbet (B) and FoxP3 (C) was measured on day 3 (n=2 indep. exp., all qPCRs performed in duplicates, values are normalized to HPRT and expressed relative to T_{H0}).

Suppl. figure 5

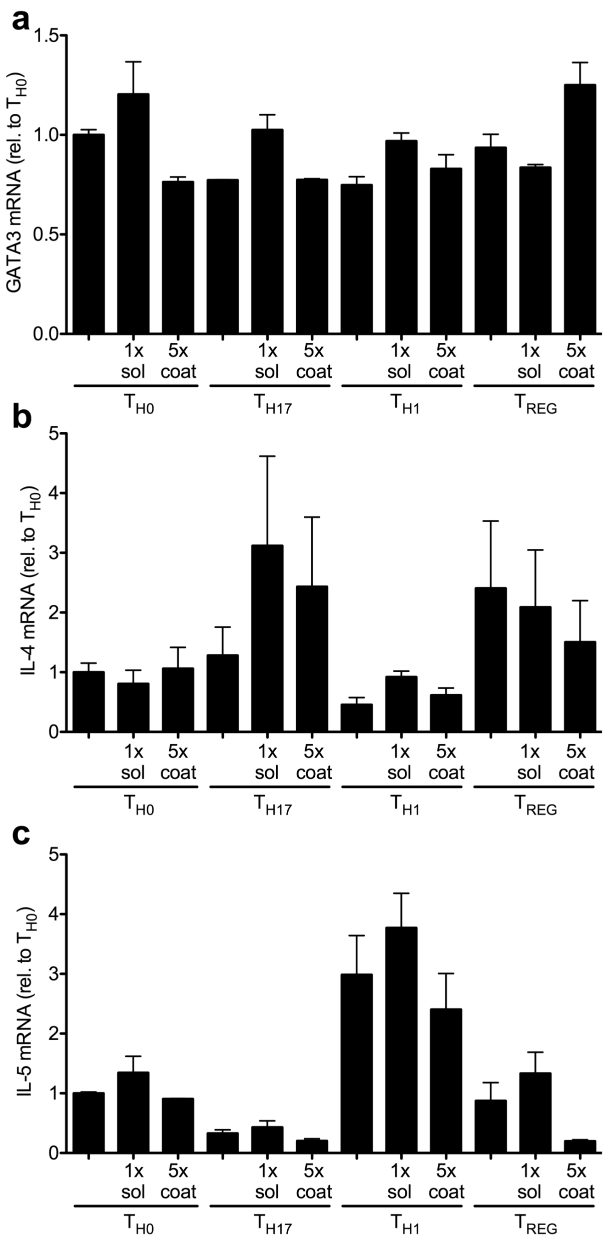

Supplemental figure 5: T_{H2} marker expression in response to coated cytokine preparations

(A-C) Fractalkine receptor deficient (*CX3CR1*$^{-/-}$) cells were subjected to T_{H17} polarization in the absence and presence of coated ("coat") and soluble ("sol") recombinant fractalkine at 100nM final concentration. mRNA expression of GATA3 (A), IL-4 (B) and IL-5 (C) was measured on day 3 (n= 2 indep. exp., all qPCR performed in duplicates, values are normalized to HPRT and expressed relative to T_{H0}).

Suppl. figure 6

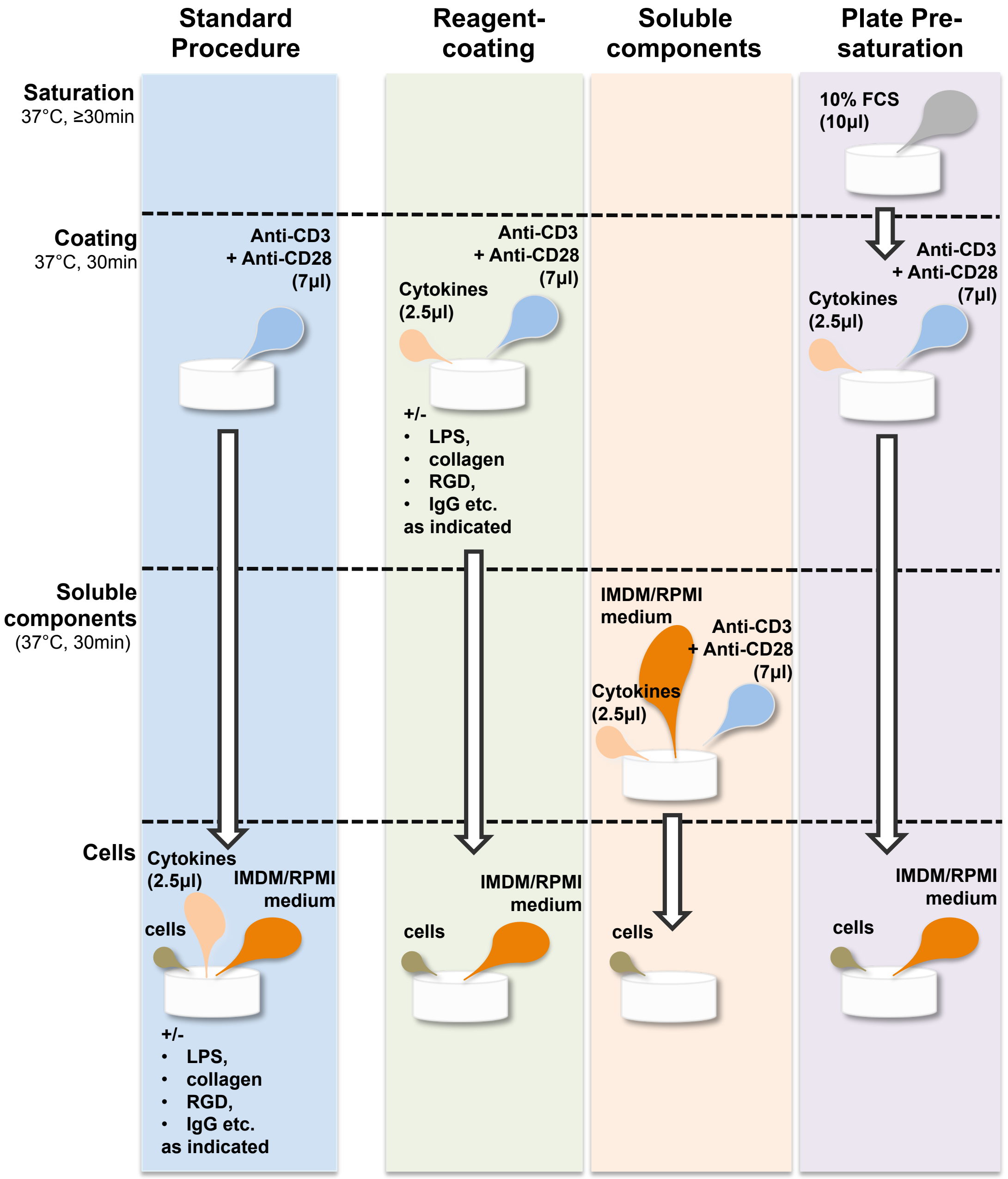

Supplemental figure 6: Schematic view of cell culture well preparation.

Sequence and timepoints of reagent addition are depicted.

Suppl. figure 7

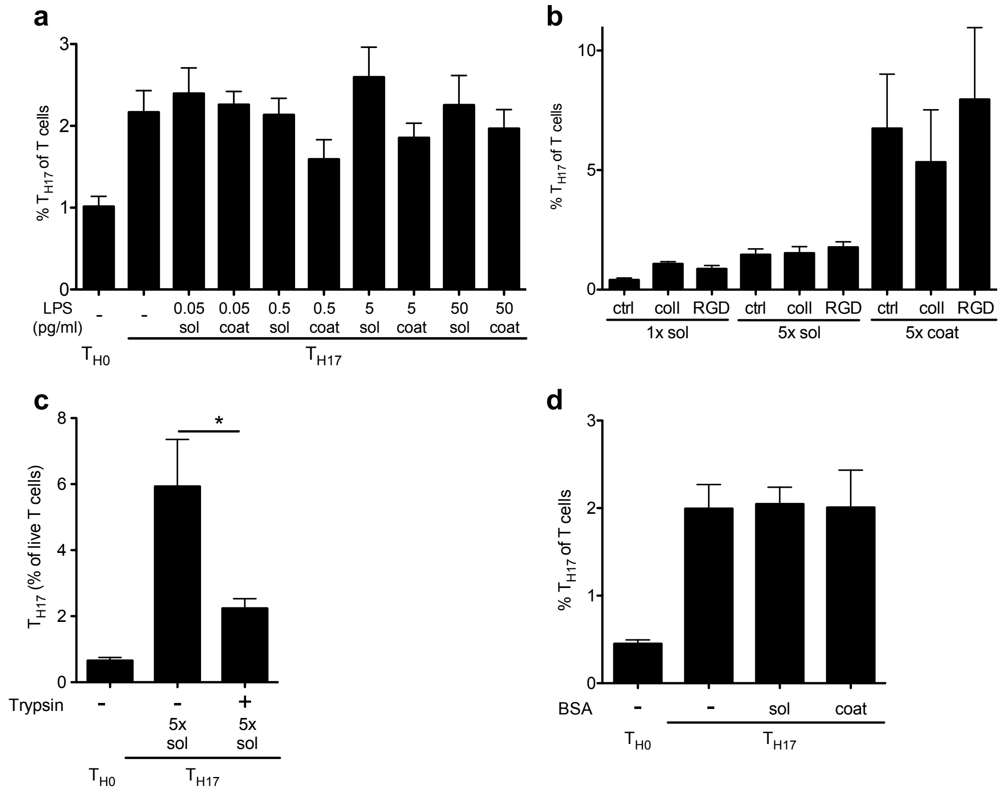

Supplemental figure 7: Effect of coating LPS and integrin receptor stimulators, efficacy of trypsin digestion and very-low dose coated BSA on T_{H17} abundance

(A) Pre-coating the cell culture plate with a range of LPS concentrations did not affect T_{H17} polarization (n=4 from 2 exp.). **(B)** Pre-coating with the integrin ligand RGD peptide did not affect the proportion of T_{H17} cells (n=4 from 2 exp.). **(C)** Protein digestion by trypsin significantly decreases specific cytokine polarizing function during T_{H17} polarization (n=4 from 2 exp.). **(D)** Pre-coating with bovine serum albumin (BSA) at a concentration corresponding to the total amount of cytokine used for coating (3μl at 10ng/μl) did not affect the proportion of T_{H17} cells (n=4 from 2 exp.). All T_{H17} polarizations were conducted for 4 days and cells restimulated with PMA/ionomycin.

Suppl. figure 8

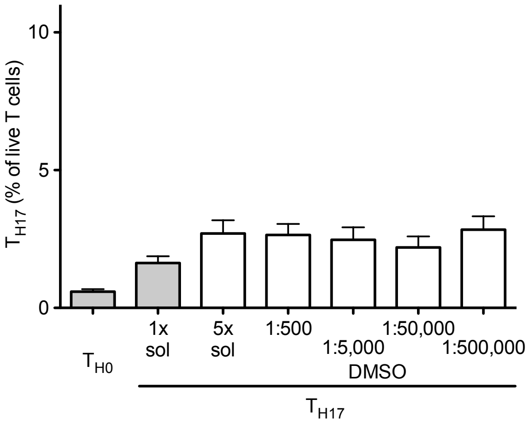

Supplemental figure 8: Effect of DMSO on T_{H17} polarization

T_{H17} polarization was conducted with DMSO in the range of concentrations used for AhR inhibitors and agonists (n=6 from 3 independent experiments conducted together with the experiments depicted in figure 3B).

Supplemental tables

Suppl. table 1: Summary of information on cytokine preparations from the manufacturers and stock solutions used

**Cytokine**	**Manu-facturer**	**Catalogue number**	**Source**	**Purity (method)**	**Endotoxin level**	**Preparation**	**Other constituents**	**Stock solution**	**Carrier protein**	**Final stock solution**
IL-2	Peprotech	212-12	E. coli	>98% (SDS-PAGE gel and HPLC)	<0.1ng/µg protein (<1EU/µg)	0.2µm filtered	Lyophilized from 10mM Na citrate, pH 4.0	1µg/µl in d H_20	No	10ng/µl in PBS, 0.1% BSA
IL-6	Biolegend	575704	E. coli	>98% (Coomassie stained SDS-PAGE)	<0.01ng/µg protein (<0.1EU/µg)	0.22µm filtered	Na Acetate, EDTA	0.2µg/µl	No	50ng/µl in PBS, 0.1% BSA
IL-12 (p70)	Biolegend	577002	Sf9 insect cells	95% (Coomassie stained SDS-PAGE)	<0.01ng/µg protein	0.22µm filtered	10 mM NaH2PO4 and 150 mM NaCl, pH 7.2	0.2µg/µl	No	20ng/µl in PBS, 0.1% BSA
IL-23	Biolegend	589002	Insect cells	>95% (Coomassie stained SDS-PAGE)	<0.01ng/µg protein	0.22µm filtered	PBS	0.1µg/µl	No	20ng/µl in PBS, 0.1% BSA
TGFβ1	Biolegend	580702	CHO cells	>98% (Coomassie stained SDS-PAGE)	<0.01ng/µg protein (<0.1EU/µg)	0.22µm filtered	30% acetonitrile, 0.1% trifluoro-acetic acid	0.2µg/µl	No	1ng/µl in PBS, 0.1% BSA
CX3CL1	R&D Systems	362-CX-025	E. coli	>97% (SDS-PAGE under reducing conditions, silver stain)	<0.01ng/µg protein	0.2µm filtered	Lyophilized in acetonitrile, trifluoro-acetic acid	0.1µg/µl in PBS	No	1ng/µl in PBS
CX3CL1	Peprotech	300-31	E. coli	>98% (SDS-PAGE gel and HPLC)	<0.1ng/µg protein (<1EU/µg)	0.2µm filtered	Lyophilized	0.1µg/µl in dH_20	No	10ng/µl in PBS, 0.1% BSA
IL-17A	R&D Systems	421-ML-025	E. coli	>97% (SDS-PAGE under reducing conditions, silver stain)	<0.01ng/µg protein	0.2µm filtered	Lyophilized in acetonitrile, trifluoro-acetic acid	0.1µg/µl in 4 mM HCl, PBS	No	1ng/µl in PBS
IL-17A	Miltenyi Biotech	130-103-448	E. coli	>97% (SDS-PAGE)	<0.1ng/µg protein (<1EU/µg)	0.2µm filtered	Lyophilized	0.1µg/µl in dH_20	No	1ng/µl in PBS, 0.1% BSA

Supplemental table 2: Protein contents of recombinant IL-17A preparations

Data for recombinant murine IL-17A from Miltenyi Biotech and R&D systems companies are available as .xls file.
